# Monthly Summary

**Published:** 1874-08

**Authors:** 


### MONTHLY SUMMARY.

*Solubility of Arsenious Acid in Alcohol.*—Liquid for the preservation of Anatomic Specimens.—By Dr. G. Mehu.—For about two years, I have prepared large quantities of an antiseptic liquid for the conservation of anatomic specimens at Necker Hospital ; after many trials, I have adopted the following formula, giving very satisfactory results.

This liquid is slightly alcoholic, therefore it does not contract soft tissues ( generally the bladders of those troubled with calculi or affections of the prostrate ) for the preservation of which it is applied ; it is sufficiently rich in arsenious acid to prevent their softening and their decomposition. Furthermore, in order to prevent more surely the development of cryptogamic vegetations so often observed in arsenical solutions weak in alcohol, I have added 1 part of crystallized phenic acid to 100 of liquid.

The preparation of this liquor led me to a practical observation, which I was far from looking for ; it is this : concentrated, boiling alcohol dissolves easily and rapidly powdered arsenious acid, and in such large quantities that I regard this mode of solution as incomparably more advantageous than the employment of boiling water. Those who have had to dissolve arsenious acid for medical use, ( Boudin's liquor and others ) know how slow that solution proceeds, and how difficult it is to make the more coarsely powdered particles disappear. The result of this observation is at present confirmed by more than fifty trials, made every time with 200 and 300 grammes of arsenious acid dissolved by alcohols of different degrees, and at different temperatures ; this investigation being much more complicated than would appear at first sight, will be the object of future study.

The substitution of an alcoholic liquid for water, as solvent, has moreover another advantage : it permits to operate in a glass capsule, over a bath of boiling water, or at a temperature near it, which avoids all attention, all loss and all projection of a dangerous liquid.

The formula for this conservator liquid is as follows :

| | | |
|---|---|---|
| Arsenious Acid, - - - | 20 | grammes. |
| Phenic Acid, cryst., - - - | 10 | " |
| Alcohol, - - - - | 300 | " |
| Distilled Water, - - - | 700 | " |

I place the arsenious acid in a glass capsule, add the larger part of the alcohol, and about a third of the water; place the capsule over a bath of boiling water, (any iron pot over an ordinary stove,) when the arsenious acid disappears promptly, only the more coarsely pulverized pieces remaining. I decant and filter the liquid, which I dilute immediately with boiling water to prevent the deposit of the arsenious acid, which would take place on cooling. The small quantity of arsenious acid remaining undissolved is added to the alcohol and water and subjected to another ebullition. To 100 parts of this arsenical liquid I add 1 part of crystallized phenic acid, previously liquified by gentle heat; I agitate the whole, in order that the solution may become homogeneous.

What has been said shows that it is advantageous to employ the arsenious acid finely pulverized, in order that the process may proceed more rapidly.—*The Pharmacist.*

---

*Nickeling.*—In answer to numerous inquiries, writes Mr. S. P. Sharpless in the *Boston Journal of Chemistry*, I again give a brief description of the process of nickeling. The patent is still before the courts, and no decision has been reached in regard to it.

The double sulphate of nickel and ammonium, which is the salt that is generally used, may now be had in commerce almost pure. It is manufactured on a large scale by Joseph Wharton, of Camden, N. J., who controls the nickel market in this country. Cast nickel plates for anodes may be obtained from the same source. The anodes should considerably exceed in size the articles to be covered with nickel. Any common form of battery may be used. Three Daniel's or Smee's cells, or two Bunsen's, connected for intensity, will be found to be sufficient. The battery power must not be too strong, or the deposited nickel will be black. A strong solution of the sulphate is made and placed in any suitable vessel; a glazed stoneware pot answers very well if the articles to be covered are small. Across the top of this are placed two heavy copper wires, to one of which the articles to be covered are suspended, to the other the anode. The wire leading from the zinc of the battery must then be connected with the wire from which the articles are suspended, the other battery wire being connected with the anode.

In order to prepare the articles for coating, they must be well cleaned by first scrubbing them with caustic soda or potash to remove any grease, and then dipping them for an instant in *aqua regia* and afterwards washing thoroughly with water, taking care that the hand does not come in contact with any part of them. This is accomplished by fastening a flexible copper wire around them, and handling them by means of it. The wire serves afterwards to suspend them in the bath.

If the articles are made of iron or steel, they must be first covered with a thin coat of copper. This is best done by the cyanide bath, which is prepared by dissolving precipitated oxide of copper in cyanide of potassium. A copper plate is used as an anode. After they are removed from the copper bath, they must be washed quickly with water and placed in the nickel bath; if allowed to dry or become tarnished, the nickel will not adhere.

Great care must be used through the whole process to keep all grease, dust, or other dirt from the articles to be covered, or else the result will be unsatisfactory. The whole process is one of the most difficult that is used in the arts, it being far easier to gild, plate, or copper an article than to nickel it; but if due care be taken, the results will amply pay for the trouble.—*Druggists' Circular.*

---

*Large Calculus.*—The following account of a calculus of gigantic magnitude is copied by a Mr. Gouge, from the preface to an old book of sermons by the Rev. Nicholas Byfield, of Isleworth, who lived in the time of Queen Elizabeth and James I. The book was published, after his death, by the editor, Mr. Gouge, to whom we are indebted for the details of this remarkable case, and is dated 1623.

"It appears that he carried a torturing stone in his bladder fifteen years together and upward. I have heard it credibly reported that, fifteen years before his death, he was by a skillful chirurgeon searched; and that, upon that search, there was a stone found to bee in his bladder; whereupon hee used such means as were prescribed to him for his case, and found such help thereby as he thought; that either the chirurgeon which searcht him was deceived; or that the means which he used had dissolved the stone. But time which manifesteth all things, shewed, that neither his chirurgeon was deceived, nor yet his stone dissolved; for, it continued to grow bigger and bigger, till at length it came to bee of an incredible greatness. After his death, hee was opened, and the stone taken out; and being weighed, found to be 33 *ounces and more in weight*; and in measure about the edge *fifteen inches and a halfe:* about the length *above* 13 *inches;* about the breadth, *almost thirteen inches;* it was of a solid substance; to look upon, like a flint. There are many eie-witnesses besides who can justifie the truth hereof. A wonderful work of God it was, that he should bee able to carry such a stone in his bladder, and withall to do the things which he did."—*British Medical Journal.*

*On Iodoform as a Topical Remedy.*—Dr. Courteaux (*Annales de Dermatologie et de Syphiligraphie*, vol. v. 1873–4) gives a summary notice of two recent works by Petiteau and Isard on the use of iodoform. Petiteau's conclusions, which, Dr. Courteaux says, agree with his own and those of several surgeons at the Midi Hospital of Paris, are as follows:—

1. Iodoform is locally anæsthetic.
2. When dusted on uncerating surfaces, it causes them to cicatrize rapidly.
3. It is most useful in small atonic wounds, or such as tend to creep or enlarge , soft chancres, suppurating buboes ; syphilitic, varicose, scrofulous, and cancerous ulcers.
4. It is the surest remedy to procure a prompt cicatrization of syphilitic ulcers of all kinds.
5. It may be applied as an ointment, or as a solution in glycerine and alcohol, and in these forms is preferable to the powder when there is abundant suppuration.
6. It should be always accompanied in syphilitic affections by internal treatment.

Petiteau attributes its good effects to the simplicity of the dressing, to its antiseptic power, and to the absorbent property it possesses as a powder, and to its giving off iodine freely.

Isard, while agreeing with many of the conclusions of Petiteau, maintains that phagedæna is not controlled by iodoform.

The results of some comparative experiments made by Mr. Berkeley Hill on soft venereal sores and on creeping tertiary syphilitic sores with iodoform and other applications, corroborate Isard's conclusion concerning the feeble power of iodoform to arrest certain obstinately spreading sores. The early venereal sores were in seventeen out of twenty cases not at all controlled by iodoform ; and, though more efficacious with tertiary ulcers, it was not uniformly effectual.—*London Med. Record.*

---

*Remedy for Toothache.*—Having spent a number of years of my professional life in the country, where no dentist was near, and therefore being often called to prescribe for toothache, I tried many combinations of drugs, and many published formulæ, with more or less satisfaction to myself and patients. But several years since I hit upon the following combination, which I have found better than any I have ever used, and it will give relief in almost all cases where such a remedy is applicable:

℞ Carbolic acid, saturated solution ; hydrate of chloral, saturated solution ; camphorated tincture of opium ; fluid extract of aconite, of each an ounce ; oil of peppermint ½ oz. M.—Apply by saturating a pledget of cotton (or preferably a small piece of sponge), and pack closely into the cavity of the decayed tooth. —*Dental Cosmos.*

*A French Charlatan.*—An irregular practitioner was recently fined at Brest, for practicing without a diploma. He was a retired naval officer who had found means, during several years, to increase his income by the sale of tiny bits of sugar steeped in liquids, for the nominal sum of fivepence each. He called himself a homœopath, and openly visited patients, and gave consultations, charging the same fees as the medical men of Brest. This was no vulgar and illiterate impostor, and his success was the greater as he was a superior naval officer, and always gave his consultations in full uniform.—*Med and Surg. Reporter.*

---

*The Use of Iodic Acid by Hypodermic Injection.*—For some time the hypodermic syringe has been used to introduce fluids into the centre of glandular tumors. Lately Dr. Luton, of the Rheims School of Medicine, has drawn attention to the remarkable success he has had in the dispersion of glandular and other tumors by the use of iodic acid thus applied. Goitres have been so treated, the solution he uses being about one-fifth acid, the balance water. A sharp local action at once occurs, but he has seen no inconvenience follow, and the resolution of the tumor has finally been effected without suppuration.—*Med Press and Circular.*

---

*The Prevention Cicatrices.*—In the Military Hospital of Constantinople, I attended a soldier cook, with an extensive scald in the third degree, on the right arm, forearm, and side. After the healing of the wound, the forearm was close to the arm in such a way that all the extension above the length of the fingers in flexion was impossible, the fingers in this position reaching the deltoid. The nodular tissue extended over four-fifths of both arm and forearm.

To overcome the difficulty of motion I have proceeded in the following manner: With a *trois-quart explorateur* I made a series of perforations through the whole length of the cicatricial tissue at the distance of two or three lines from each other. In each of these holes I introduced a silver wire of a good size, and left it in place. When those openings were healed, I made an incision through the whole cicatrix, following the line of the artificial openings. I placed the arm in extension, where it healed, and I obtained a complete success.—*Dr. Andre's Clinic.*

---

*Death from a Dose of Chloral and Bromide of Potassium.*—The Cincinnati *Clinic* reports the death of a physian of that place from having taken at one dose a mixture containing one-half oz. of bromide of potassium, and one drachm of chloral.

*Bone and Skin Grafting.*—M. Howse, recommends in cases where suitable grafts cannot be obtained from human sources, to use the mucous membrane from the lips of guinea-pigs or rabbits; he has succeeded in such cases, and advises that rabbits fresh from the country pastures be selected. Not only have skin grafts from animals succeeded, but M. Reverdin gives an illustration of the case of a Russian gentleman from whose cranium a Tartar had sliced a piece by a sabre cut; the loss was supplied by a fresh piece from the skull of a dog, and the graft succeeded completely.—*L' Union Medicale—The Clinic.*

---

*Trichinæ.*—At Harrisburg, Pa., June 1, a post-mortem examination was made of the body of George Cordes, who died from eating ham containing trichinæ. Under the microscope thousands of trichinæ were visible, sixty-five being counted in the space of an eighth of an inch; and the whole body doubtless contained millions. Cordes suffered terribly before death.—*Medical and Surgical Reporter.*

---

*Death from Lancing of the Gum.*—In the *American Medical Journal*, for April, are given the particulars of the death of a child fourteen months old, from hæmorrhage occasioned by the lancing of the gum over a molar tooth. The blood oozed from the divided gum for three days, in spite of all efforts to suppress it. The child was well developed, and healthy from birth, and no previous suspicions had been entertained of the existence of a hæmorrhagic diathesis.—*Boston Med. and Surg. Journal.*

---

*Lusus Naturæ*—Dr. W. H. Bent, of Argyle, N. S., reports the following peculiar case of monstrosity occuring in a child whose parents reside in Pubnico. It has three legs in perfect use, two complete sets of sexual organs (male); two perfect arms, and two imperfect ones—the latter growing from the middle of the back and curving in a line with the lower ribs. The head is finely developed, and what appears to be a second one is situated half way down the left side of the neck. It is considered a great wonder among the French Acadians.—*London Medical Record.*

---

*Methyl-bichloride.*—This anæsthetic is being used at the St. John's hospital with the best of results, and so far it seems to possess many advantages over both chloroform and ether.—*Mo. Clin. Record.*

